# Astrocytes in mouse models of tauopathies acquire early deficits and lose neurosupportive functions

**DOI:** 10.1186/s40478-017-0478-9

**Published:** 2017-11-29

**Authors:** Marta Sidoryk-Wegrzynowicz, Yannick N. Gerber, Miriam Ries, Magdalena Sastre, Aviva M. Tolkovsky, Maria Grazia Spillantini

**Affiliations:** 1 Department of Clinical Neurosciences, University of Cambridge, The Clifford Allbutt Building, Cambridge, CB2 0AH UK; 2 Division of Brain Sciences, Department of Medicine, Imperial College London, London, UK

**Keywords:** Astrocyte conditioned medium, Frontotemporal dementia, Neurotoxicity, Neuroprotection, Synaptic markers, Tau, TSP-1

## Abstract

Microtubule-associated protein tau aggregates constitute the characteristic neuropathological features of several neurodegenerative diseases grouped under the name of tauopathies. It is now clear that the process of tau aggregation is associated with neurodegeneration. Several transgenic tau mouse models have been developed where tau progressively aggregates, causing neuronal death. Previously we have shown that transplantation of astrocytes in P301S tau transgenic mice rescues cortical neuron death, implying that the endogenous astrocytes are deficient in survival support. We now show that the gliosis markers Glial fibrillary acidic protein (GFAP) and S100 calcium-binding protein B (S100β) are elevated in brains from P301S tau mice compared to control C57Bl/6 mice whereas the expression of proteins involved in glutamine/glutamate metabolism are reduced, pointing to a functional deficit. To test whether astrocytes from P301S mice are intrinsically deficient, we co-cultured astrocytes and neurons from control and P301S mice. Significantly more C57-derived and P301S-derived neurons survived when cells were cultured with C57-derived astrocytes or astrocyte conditioned medium (C57ACM) than with P301S-derived astrocytes or astrocyte conditioned medium (P301SACM), or ACM from P301L tau mice, where the transgene is also specifically expressed in neurons. The astrocytic alterations developed in mice during the first postnatal week of life. In addition, P301SACM significantly decreased presynaptic (synaptophysin, SNP) and postsynaptic (postsynaptic density protein 95, PSD95) protein expression in cortical neuron cultures whereas C57ACM enhanced these markers. Since thrombospondin 1 (TSP-1) is a major survival and synaptogenic factor, we examined whether TSP-1 is deficient in P301S mouse brains and ACM. Significantly less TSP-1 was expressed in the brains of P301S tau mice or produced by P301S-derived astrocytes, whereas supplementation of P301SACM with TSP-1 increased its neurosupportive capacity. Our results demonstrate that P301S-derived astrocytes acquire an early functional deficiency that may explain in part the loss of cortical neurons in the P301S tau mice.

**Electronic supplementary material:**

The online version of this article (10.1186/s40478-017-0478-9) contains supplementary material, which is available to authorized users.

## Introduction

Several neurodegenerative diseases, such as Alzheimer’s disease (AD), progressive supranuclear palsy (PSP), corticobasal degeneration (CBD), Pick’s disease (PiD), argyrophilic grain disease, and inherited frontotemporal dementia and parkinsonism linked to chromosome 17 (FTDP-17T) are characterized by the presence of abnormal intracellular filamentous protein inclusions that consist of hyperphosphorylated microtubule-associated protein tau and are collectively designated as tauopathies [18, 26, 43]. The identification of mutations in the MAPT gene in FTDP-17T [22, 43, 44] has established that dysfunction or misregulation of the tau protein is central to the neurodegenerative process in disorders with tau pathology. Furthermore, in AD it is the accumulation and dysfunction of tau that causes cell death and best correlates with the appearance of dementia [7, 18].

Despite the knowledge that the presence of misfolded hyperphosphorylated tau is critical for development of disease and neuronal death [15, 36], the mechanism of tau-related toxicity is still not clear. P301S tau transgenic mice (P301S mice) expressing human tau under the control of the neuronal Thy1.2 promoter develop neuronal tau aggregates in many brain areas [1]. Tau pathology develops stereotypically between 2 and 5 months of age culminating in neuronal death most notably observed in the superficial layers of the motor and, perirhinal and piriform cortices [1, 9, 51, 52]. To determine whether altering the environment can extend neuronal survival, we transplanted neuron precursor cell (NPC)-derived astrocytes and showed that neuronal death in the superficial layers of the motor cortex was prevented [19], indicating a deficiency in survival support, or a gain of toxic functions, by the endogenous astrocytes. Astrocyte activation and reactive gliosis are associated with disease progression in almost all human neurodegenerative diseases [33, 48] and astrogliosis appears to precede neuronal loss, suggesting an important causative role of astrocytes in the development of disease [27].

Here we investigate the reasons why astrocytes from P301S mice do not prevent neuronal death whereas transplanted control astrocytes do. We show that astrocytes derived from the superficial cortex of P301S mice exhibit changes in cell specific markers that indicate astrocyte dysfunction. Moreover we demonstrate in in vitro systems that astrocytes or astrocyte conditioned medium from wild type mice have neuroprotective and synaptogenic functions that are absent in astrocytes from P301S- or P301L-tau expressing mice, which can be attributed in part to a reduction in thrombospondin-1 (TSP-1) expression in conditioned medium from P301S astrocytes.

Overall, our data demonstrate that astrocytes in the P301S tau mice are directly involved in neuronal death even though they do not express tau, highlighting a novel important contribution of astrocytes to tau-related pathogenicity, opening up new therapeutic avenues for treating diseases with tau pathology.

## Materials and methods

### Animals

Neurons and astrocytes were prepared from postnatal day 1–2, or 7–9 P301S tau or P301L tau female and male mice [1, 45] along with age-matched C57BL/6 control mice. The tau mutation in the P301S mice is in the human 0N4R isoform whereas in the P301L mice, it is in the 2N4R isoform. Brain extracts were prepared from 3 to 5 month-old P301S and C57BL/6 mice. This research was conducted under the Animals (Scientific Procedures) Act 1986, Amendment Regulations 2012, following ethical review by the University of Cambridge Animal Welfare and Ethical Review Body (AWERB).

### Brain extracts

Mice were killed by cervical dislocation and brains were snap frozen on dry ice. Thick coronal slices (100 μm) extending from approximately 2.2 mm rostral to the bregma to the bregma were cut using a cryostat. The upper layers of the sensorimotor cortex were specifically dissected out using an ophthalmic blade. Dissected brain tissues were stored at −80 °C until use.

### Astrocyte cultures

Primary astrocyte cultures were prepared from the cerebral cortex of 1–2 or 7–9 day-old C57 and P301S mice, or 7–8 day-old P301L mice as described previously [42]. Briefly, mice were decapitated, the cortex was isolated and was triturated in HBSS (Hanks’ Balanced Salt Solution) by pipetting up and down. The cell suspension was incubated in 0.05% trypsin in HBSS at 37^•^C to further dissociate the cells. After 30 min, fetal bovine serum (FBS) was added to a final concentration of 5% and the cell suspension was centrifuged at 1200 rpm. Pelleted cells were resuspended in DMEM with Earle’s salts supplemented with 10% FBS, 100 units/mL of penicillin and 100 μg/mL of streptomycin and plated in non-coated T75 flasks (ThermoScientific) at a density of 10^5^ cells/ml. The cultures were maintained at 37 °C in 5% CO_2_. Twenty-four hours after the initial plating, the medium was changed to remove non-adherent cells. When cultures reached confluence (about 1 week), non-astrocytic cells were separated from astrocytes by shaking for 15 h at 50 rpm at 37 °C (Luckham R300). Astrocyte-enriched cultures were then passaged into PDL-coated plates and maintained under the same conditions as the initial cultures. The surface-adherent monolayer cultures were > 98% positive for the astrocytic marker glial fibrillary acidic protein (GFAP). Cells were used for experiments after 5–6 days.

### Neuronal cultures

Primary neuronal cultures were prepared from the cerebral cortex (3 brains per preparation) of ≥7 day-old or 1–2 day-old C57 and P301S mice. Briefly, neurons were isolated following the same protocol used for astrocytes and cultured in Neurobasal medium supplemented with 5% heat-inactivated bovine calf serum (Hyclone), B27, 1 mM L-glutamine, 100 U/mL of penicillin and 0.1 mg/mL streptomycin. Neurons were plated at a density of 10^5^ cells/ml on 35 mm dishes coated with poly-D-lysine (10 μg/ml; Sigma). Cytosine arabinoside (2.5 μM) was added to the cultures on the second day after seeding to inhibit the proliferation of non-neuronal cells. Cells were used for experiments after 5–6 days. This protocol produced a neuron-enriched culture (95% of neurons).

### Direct neuron–astrocyte co-cultures

Primary purified astrocytes from the second passage were plated at a density of 1.7 × 10^4^ cells/cm^2^ on the top of AraC-treated primary neurons that had been in culture for 5–7 days. Co-cultures were fed with a mixture of one-third of astrocytic and two-thirds of neuronal medium, maintained at 37 °C in a humidified atmosphere of 5% CO_2_ and analyzed 4 and 8 days later. Cells were fixed and stained with the neuronal marker β-III-tubulin and the astrocytic marker GFAP to determine neuron/astrocyte number. Several fields per each experimental condition were scored for presence of neurons and astrocytes as described in the figure legends and the total number counted was used as a single value for statistical purposes. Results were obtained from 3 to 4 independent experiments (cell cultures) and each culture contained cells from the cortex of three mice.

### Astrocyte conditioned medium (ACM)

Pure astrocyte cultures grown as described above for 5–6 days were passaged once. After reaching confluence, cultures were thoroughly washed to eliminate residual serum, and the cultures were maintained without fetal bovine serum for 1 day. The medium was then collected and centrifuged to remove cellular debris at 1000 rpm for 5 min and used immediately. To analyse the effect of ACM on neuronal survival, the medium in which the neurons were grown for 5–7 days was replaced with ACM and survival was analyzed after 4 and 8 days by counting β-III-tubulin positive neurons.

### TSP-1 withdrawal or supplementation to ACM

TSP-1 was depleted from C57ACM by immunoprecipitation with an anti-TSP-1 antibody (Abcam, ab140250, 1:500) using magnetic Protein G Dynabeads (Invitrogen). Briefly, anti-TSP-1 antibody (Abcam 140250, 1:500) was incubated with Dynabeads with rotation for 10 min at room temperature. Then, C57ACM was added to the Dynabead-Ab complex, rotated for 10 min at room temperature, and immune complexes bound to the beads were pelleted by applying a magnetic field. The TSP-1-depleted ACM supernatant was collected and applied to neurons for 4 days. TSP-1 removal was verified by immunoblotting. For TSP-1 supplementation, ACM from P301SA was enriched with recombinant mouse TSP-1 (rTSP-1, 500 ng/ml, NovusBio) and the mixtures were added to cultured neurons for 4 days. Neuronal survival was determined by counting neurons identified by immunocytochemistry with anti-β-III-tubulin.

### Proliferation capacity

Astrocytes grown to 98% confluence, were repassaged and analyzed after 2 days. Cells were incubated with the thymidine analogue 5-ethynyl-2′-deoxyuridine (EdU, 10 μM final concentration, ThermoScientific) for 2 h at 37 °C, fixed and stained using the Click-iT® EdU Alexa 488 Cell Proliferation kit (ThermoScientific).

### Western blot analysis

Tissue, cultured astrocytes or neurons were lysed in RIPA buffer (150 mM NaCl, 1.0% IGEPAL® CA-630, 0.5% sodium deoxycholate, 0.1% sodium dodecyl sulfate and 50 mM Tris, pH 8.0) containing protease and phosphatase inhibitor cocktails (Sigma). Tissue was left in RIPA buffer for 20 min on ice before homogenisation with a teflon pestle. Homogenates were spun at 13,000 × g for 30 min and the supernatants were used for analysis. ACMs were concentrated by spinning at 3750 × g for 25 min in an Amicon centrifuge filter tubes with a 10 kDa molecular weight cut-off. Protein concentrations in the tissue extracts, cell lysates or ACM were determined with the bicinchoninic acid (BCA) protein assay kit (Pierce, ThermoScientific). An equal amount of protein from cells or ACM (15 μg) was loaded and run on a 12% SDS-PAGE and then transferred to a polyvinylidenefluoride membrane (EMDMillipore). Non specific background was blocked by a 1 h incubation at room temperature in 5% non-fat dry milk in Tris Buffered Saline with 0.1% Tween 20 (TBS-T). Incubations with primary antibodies were carried out at 4 °C for 24 h in 5% non-fat milk in TBS-T buffer at the following antibody concentrations: anti-GLAST (Abcam, ab416, 1:1000), anti-GLT1 (Abcam, ab41621, 1:1000), anti-GS (Abcam, ab49873, 1:2000), anti-GFAP (Abcam, ab10062, 1:2000), anti-S100β (Abcam, ab14688, 1:1000), anti-TSP-1 (Abcam, ab85762, 1:1000), anti-PSD-95 (Abcam, ab18258, 1:2000), anti-synaptophysin (SNP) (Abcam, ab106618, 1:1000), anti-beta actin (Sigma, A2066, 1:5000). Secondary antibody incubations were performed at room temperature for 1.5 h using HRP-conjugated anti-rabbit IgG (ThermoScientific, 1:2000) or anti-mouse IgG (Sigma, 1:4000). For ACMs, blots were visualized with Ponceau S (Sigma) and developed with Supersignal West Dura Extended Duration Chemiluminescent Substrate (Pierce, ThermoScientific).

### Immunocytochemistry

Primary neuronal, astrocytes or astrocytic-neuronal co-cultures plated on glass coverslips were washed twice with TBS and fixed at room temperature for 10 min with 100% cold methanol. Cells were permeabilized with 0.1% Triton X-100 in PBS for 15 min and then incubated for 1 h in 5% goat serum to reduce nonspecific background. After an overnight incubation at 4 °C with the primary antibodies: [chicken or mouse anti-glial fibrillary acidic protein (Abcam, ab4674, 1: 200 or Dako, z0334, 1:500), anti-β-III-tubulin, (Abcam, ab18207, 1:500 or Covance, MMS-435P 1:1000), anti-synaptophysin (Abcam, ab106618, 1:500), anti-NeuN (Millipore, MAB377, 1:500)], cells were washed with TBS and incubated with secondary AlexaFluor-conjugated antibodies appropriate for the species (Molecular Probes, 1:500). To visualize cell nuclei, cultures were rinsed and then incubated in 4′,6-diamidino-2-phenylindole dihydrochloride hydrate (DAPI)/antifade (Sigma, 1:1000) diluted in TBS or Hoescht dye (Sigma, 1:5000) for 10 min at room temperature. Cover slips were mounted in FluorSave™ (EMD Millipore) and pictures were taken with a wide-field fluorescence microscope (Leica DMI 4000B microscope using a Leica DFC3000 G camera and the Leica application suite 4.0.0.11706).

### Image analysis

Western blot and SNP intensity analyses were carried out using ImageJ (Rasband, W.S., ImageJ, U.S. National Institutes of Health, http://imagej.nih.gov/ij/, 1997–2014). Quantification of SNP expression in neurons was performed by measuring mean fluorescence staining intensity within the contour drawn around the individual cells in SNP-stained neuronal cultures. At least six cells per field, and four fields per technical replicate were analyzed. Bands on blots were quantified by measuring mean gray value of individual bands using the Measure tool in ImageJ or the AlphaEaseFC Imaging System software (Alpha Innotech).

### Statistical analysis

Data are expressed as mean ± SEM. Results from technical replicates or from counting several fields in each culture were pooled to give a single value for statistical purposes. Statistical analyses for significant differences were performed with unpaired *t* test, or one- or two-way ANOVA followed by Tukey’s posthoc test or Mann-Whitney where appropriate, using GraphPad Prism 5.0 software. The criterion for statistical significance was *p* < 0.05.

## Results

### Astrocytes from P301S mice exhibit loss of functional proteins and gain of gliosis markers

**Fig. 1:**
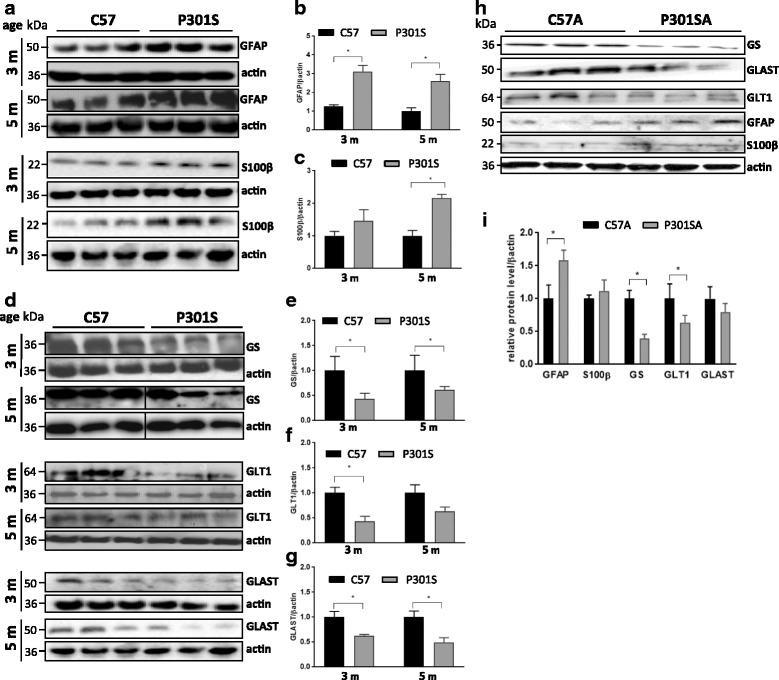
Astrocytes from P301S mice express more GFAP and S100β and less GS, GLT-1 and GLAST than astrocytes from control mice. **a**, **d** Representative blots of astrocyte-specific protein markers related to gliosis/proliferation (GFAP, S100β) and function (glutamine synthetase (GS) and the glutamate transporters (GLT-1 and GLAST)) in the superficial motor cortex of 3 month-old and 5 month-old C57 and P301S mice. **h** Expression of the same markers in primary pure cultured astrocytes from 7 day-old mice after days 8 in vitro (98% culture confluence). Mean ± SEM, *p < 0.05 vs control; unpaired *t* test, *N* = 3 independent experiments (mice: GFAP, S100β (**b**, **c**); GLT-1, GLAST, GS (**e**–**g**); primary cultures **i**). Vertical lines in (**d**) denote point were the picture of the Western blot was assembled from two parts cut from the same blot

To examine whether astrocytes had altered phenotypes in P301S mice at early and late stages of tau pathology, extracts from the superficial layers of the cortex of 3 and 5 month-old C57 control and P301S mice were analysed by immunoblotting. Figure 1a–c shows that lysates from P301S mice expressed higher amounts of GFAP already at 3 months of age (~3 fold), which persisted up to 5 months of age, whereas S100β expression was elevated (~2 fold in the sample from 5 month-old mice, indicating astrogliosis. In contrast (Fig. 1d–g), there was a reduction in the expression of proteins involved in key astrocytic neurosupportive functions relating to glutamine/glutamate metabolism [35], including GS (glutamine synthetase, 2 fold at 3 months (m) and 1.8 fold at 5 m); GLAST (the GLutamate ASpartate Transporter also known as EAAT1 or SLC1A3; 2 fold at 3 m and 1.5 fold at 5 m); GLT1 (glial glutamate transporter also known as EAAT2 or SLC1A2; 1.8 fold at 3 m). To determine whether astrocytes from P301S tau mice recapitulate these abnormal phenotypes in vitro, we examined the expression of the same proteins in astrocyte cultures derived from the cerebral cortices of 7–8 day-old C57 (control; C57A) or P301S tau (P301SA) mice. Astrocytes were grown to confluence, shaken to remove non-adherent cells, and re-plated to generate 98% pure astrocytic cultures before protein extraction. Similar to the results obtained from the brains, GFAP protein expression was significantly elevated, while those of GS, and GLT1 were significantly decreased in lysates from P301SA compared to C57A (Fig. 1h, i). These results show that both cultured and endogenous astrocytes from P301S tau mice possess an abnormal phenotype from an early postnatal age that is retained in adulthood**.**

### P301S astrocytes show an increased proliferation capacity

**Fig. 2:**
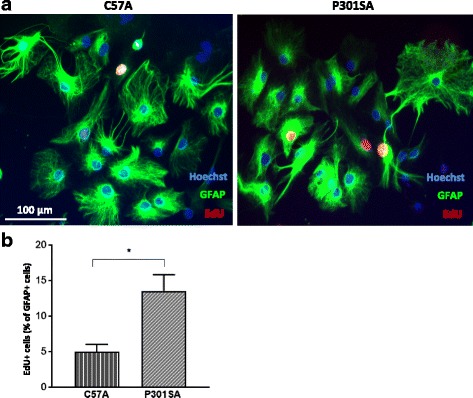
Astrocytes from P301S mice are more proliferative. Proliferation assay using EdU was performed 1 day after passage of confluent astrocyte cultures from 7 day-old pups. A higher proliferation capacity was observed in P301SA compared to C57A astrocytes. **a** Representative images where red indicates nuclei undergoing proliferation. **b** Quantification of proliferating cells, mean ± SEM, *p < 0.05 vs control; statistical analysis was performed using unpaired *t* test. *N* = 3 independent experiments where counting from three technical replicates (wells) in which at least three fields per well were analyzed constitute one value for statistical purposes. EdU, 5-ethynyl-2'-deoxyuridine

Astrocyte proliferation is a prominent cellular response to diverse brain pathologies, which induce heterogeneous and progressive changes in astrocyte gene expression and cell function. Having observed significant increases in expression of GFAP in P301SA, we examined the proliferation of the cultured astrocytes over 24 h by incorporating the alkyne-modified thymidine analogue EdU into the DNA of dividing cells followed by labelling with AlexaFluor488 using click chemistry. Consistent with the observed increase in GFAP, there was a significant increase in the rate of proliferation of P301SA compared to control C57A (Fig. 2a, b).

### Effect of astrocyte-neuron co-cultures on neuronal survival

**Fig. 3:**
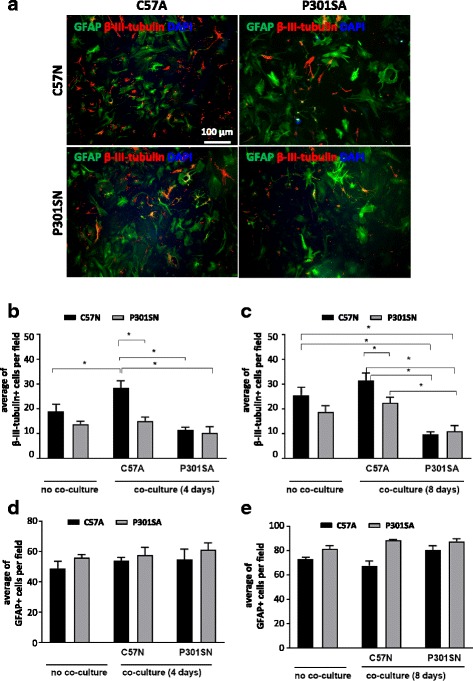
Astrocytes from P301S mice have a reduced capacity to support neuronal survival*.* Primary astrocytes (C57A and P301SA) cultured from cerebral cortex of 7 day-old mice (98% purity) were plated on top of primary neurons cultured from mice of similar age and brain region for 4–5 days. Co-cultures were maintained for 4 and 8 days. **a** Representative images of co-cultures immunostained for β-III-tubulin (red), GFAP (green) and Dapi (blue). Quantification of neuron (**b**, **c**) and astrocyte (**d**, **e**) numbers after 4 and 8 days of co-culture. Each experiment consisted of six technical replicates (wells) in which at least five fields were analyzed. Data show mean per field ± SEM from at least four independent experiments. Data were analysed using ANOVA followed by Tukey’s multiple comparison test; *p < 0.05 for these comparisons: C57N vs C57N + P301SA; C57N vs P301SN + P301SA; C57N vs C57N + C57A; C57N + C57A vs P301SN + C57A; C57N + C57A vs C57N + P301SA; C57N + C57A vs P301SN + P301SA; P301SN + C57A vs P301SN + P301SA; ANOVA of results from 4 day co-cultures revealed a significant interaction between genotype and co-culture condition [F (2, 21) = 4.477; *p* = 0.0240], significant effects of co-culture type [F (2, 21) = 14.27; *p* = 0.0001] and genotype [F (1, 21) = 14.8; *p* = 0.0009]. In 8 day of co-cultures, ANOVA revealed no interaction between genotype and culture conditions [F (2, 22) = 3.048; *p* = 0.0678], the significant effect being co-culture type [F (2, 22) = 17.51; *p* < 0.0001] and co-culture condition [F (1, 22) = 6.54; *p* = 0.0180]. No significant differences in the numbers of astrocytes were present between the various cultures

To determine whether the neuroprotective/neurotoxic effects of C57A/P301SA observed in P301S tau transgenic mice was recapitulated in vitro, we established primary co-cultures of astrocytes with cortical neurons obtained from at least 7 day-old pups, a stage at which there is consistent neuronal transgenic tau expression and initial signs of behavioural dysfunction in the P301S mice [40]. Counting the number of neurons after 4 or 8 days using anti-β-III-tubulin, and astrocytes using anti-GFAP (representative images shown in Fig. 3a), showed that there were significantly higher numbers of neurons cultured from C57 mice (C57N) or P301S tau mice (P301SN) when cells were co-cultured with C57A than with P301SA, especially notable after 8 days in culture (Fig. 3b, c). The number of astrocytes instead remained similar in all co-culture combinations (Fig. 3d, e).

### Conditioned medium from C57A and P301SA cultures replicate the effects of the respective astrocytes on neuron survival

**Fig. 4:**
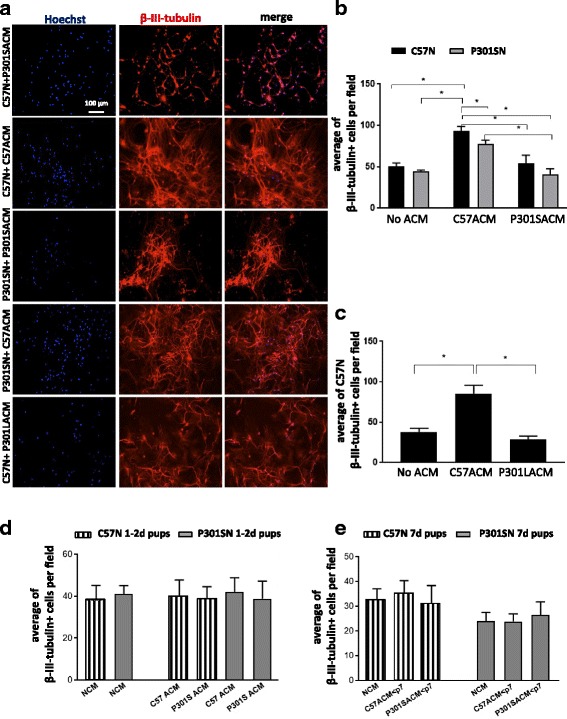
Astrocytes from P301S and P301L tau mice develop a reduced capacity to support neuronal survival during the first postnatal week. Serum-free medium conditioned by pure astrocytes derived from ≥7 day-old C57, P301S and P301L tau mice over 24 h was centrifuged to remove cellular debris and immediately added to 7 day cultured neurons extracted from 7 day-old mice. After 8 days, cells were fixed, stained with β-III-tubulin and counted. Mean ± SEM of four independent experiments where a single value is from four technical replicates (wells) in which at least five fields per well were analyzed. **a** Images of neurons treated with the various ACMs as indicated. **b** ACM from 7 day-old C57 and P301S mice; *p < 0.05 for these comparisons: no ACM C57N vs C57N + C57ACM; no ACM P301SN vs C57N + C57ACM; C57N + C57ACM vs P301SN + C57ACM, C57N + C57ACM vs C57N + P301SACM, C57N + C57ACM vs P301SN + P301SACM, P301SN + C57ACM vs P301SN + P301SACM. **c** ACM from 7 day-old C57A and P301LA; *p < 0.05 for these comparisons: no ACM C57N vs C57N + C57ACM; C57N + C57ACM vs C57N + P301LACM; Tukey’s multiple comparisons test. For 8 day of cultures, ANOVA revealed no interaction between genotype and culture conditions [F (2, 18) = 1.174; *p* = 0.3317], significant effect of culture condition [F (2, 18) = 19.73; *p* = 0.0001] and significant effect of genotype [F (1, 18) = 8.725; *p* = 0.0085]. **d** Neurons from 1 to 2 day-old mice were cultured for 4 days after which ACM derived from astrocytes cultured from 1 to 2 day-old mice was added for 4 days. **e** Neurons from 7 day-old mice were cultured for 4 days after which ACM derived from astrocytes cultured from 1 to 2 day-old mice was added for 4 days. Note that in both cases, there is no difference between the effects of ACMs from C57 or P301S mice. Mean ± SEM from three independent experiments; each value was obtained from four technical replicates (wells) in which at least five fields were examined. Values were analysed by the Mann–Whitney test

To determine whether the effect of the astrocytes on neuron survival requires constant neuron-astrocyte contact or consists of soluble factors released by the astrocytes, we cultured C57N and P301SN in astrocyte-conditioned medium (ACM) from C57 (C57ACM) or P301S (P301SACM) mice. ACMs were collected after 24 h from pure astrocyte cultures that were washed and maintained in serum-free medium. Figure 4a shows representative images of the various cultures while Fig. 4b shows that the number of surviving C57N and P301SN was significantly higher when cells were cultured with C57ACM compared to P301SACM, suggesting that C57-derived but not P301S-derived astrocytes support neuronal survival by releasing soluble factors. To exclude the possibility that the lack of survival support by P301SA is specific to the P301S tau mouse model, we generated ACM from astrocytes derived from P301L transgenic mice expressing human 2N4R tau in neurons under the Thy1.2 specific neuronal promoter [45]. These mice were chosen because the transgene is expressed under the same Thy1.2 promoter used to generate the P301S tau mice and, like the latter, have no transgene expression in astrocytes, which could confound the results (see Additional file 1: Figure S1 for evidence that no tau transgene is expressed in astrocytes in P301S tau brains or in astrocyte extracts cultured from P301S or P301L mice). Figure 4c shows that addition of P301LACM also failed to enhance neuron survival, showing that the lack of survival support by P301SA is not related to the insertion site of the transgene in the mouse genome, and can be generalized to include another transgenic model of tau pathology.

Although neither transgenic tau nor endogenous tau is expressed in astrocytes in the P301S/L mice, we asked whether there is an age dependent component to the acquisition of astrocyte dysfunction. The earliest signs of tau-induced abnormalities appear in the P301S tau mice around 3 days postnatally [40]. We therefore examined whether ACM obtained from astrocytes from 1 to 2 day old mice would have the same effect on neurons from either 1–2 day - or 7 day-old pups. Figure 4d shows that there were no differences in neuronal survival over 4 days when C57N or P301SN from 1 to 2 day-old mice were exposed to C57ACM or P301SACM that were grown from 1 to 2 day-old mice, suggesting that astrocytes acquire differential properties once pathological tau begins to be consistently present in the neurons. Moreover, neuron survival was not differentially affected after exposure of neurons derived from 7 day-old mice to ACMs from 1 to 2 day-old mice (Fig. 4e), indicating that the lack of response to ACM from 7 day-old mice in Fig. 4d was not due to the neurons being cultured from young mice. These data indicate that a certain amount of transgenic tau in young neurons is required to alter the propensity of astrocytes to support the neurons.

### P301SACM fails to support the development of synaptic protein expression

**Fig. 5:**
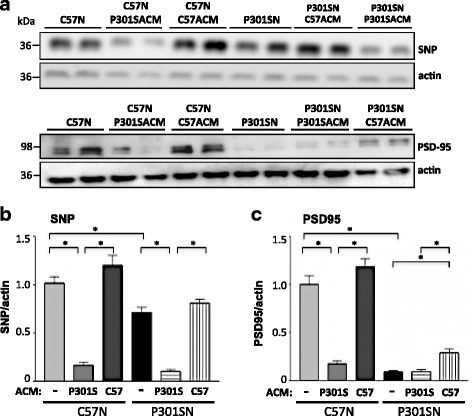
ACM from P301SA reduces synaptic protein expression in cultured neurons. C57N or P301SN cultures were exposed to C57ACM or P301SACM for 8 days after which cell lysates were analysed by immunoblotting. **a** Representative immunoblot of synaptophysin (SNP) and PSD95 in neuronal cultures with and without ACM exposure. Note the significant decline of both (**b**) SNP and (**c**) PSD95 when C57N or P301SN were cultured with P301SACM compared to neurons maintained in C57ACM. Data were normalised to β actin and represent mean ± SEM of three independent experiments performed in triplicate. *p < 0.05 for these comparisons: C57N vs C57N + P301SACM; C57N + P301SACM vs C57N + C57ACM; P301SN vs P301SN + P301SACM; P301SN vs P301SN + C57ACM; Tukey’s multiple comparisons test both for SNP and PSD95. ANOVA for SNP values revealed a significant interaction between genotype and culture condition [F (2, 12) = 29.88; *p* = 0.0001], significant effect of genotype [F (1, 12) = 307.2; *p* = 0.0001] and significant effect of culture treatment [F (2, 12) = 34.68; *p* = 0.0001]. ANOVA for PSD95 values revealed a significant interaction between genotype and culture condition (ACM) [F (2, 12) = 18.08; *p* = 0.0002], significant effect of genotype [F (1, 12) = 112.2; *p* = 0.0001] and culture treatments [F (2, 12) = 37.01; *p* = 0.0001]

Recent evidence suggests that astrocytes mediate neuroprotection by releasing factors that regulate synapse formation and integrity (for example, [46]). To address whether synaptic development is differentially affected by the two types of astrocytes, C57N and P301SN from 7 day-old pups were grown with C57ACM or P301SACM for 8 days, after which the expression of the presynaptic protein synaptophysin (SNP) and the postsynaptic protein PSD-95 were examined by immunoblotting. P301SACM significantly inhibited the expression of SNP in both C57N and P301SN and also inhibited the expression of PSD-95 in C57N (Fig. 5a–c) whereas C57ACM maintained robust SNP and PSD-95 expression, and even increased expression of PSD-95 in P301SN, where basal expression was low. This 3–4-fold reduction was not due to neuronal cell death because the percentage of death in cultures treated with P301SACM did not exceed 10%. Together our results suggest that ACM from C57 mice has beneficial effects on synaptogenesis whereas P301SACM from P301S mice older than 7 days has negative effects.

### Astrocyte protein secretome characterization

**Fig. 6:**
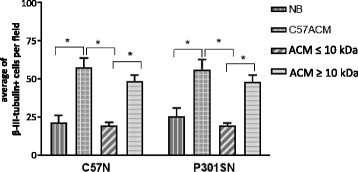
The active components in C57ACM are macromolecules with a MW above 10 kDa. C57N and P301SN cultures were exposed to complete C57ACM and the same ACM fractionated through a filter with a cut off of ≥10 kDa. The fraction ≥10 kDa (which was diluted to the original volume to offset changes due to concentrating the ACM) or ≤10 kDa was added to the neurons for 8 days. Neurons were counted after immunostaining with the neuronal marker β-III-tubulin. *p < 0.05 for comparisons between number of neurons in: NB vs C57ACM; C57ACM vs C57ACM-10 kDa; C57ACM-10 kDa vs C57ACM + 10 kDa; similar significance was found when C57N or P301SN were treated, statistical analyses was done using Tukey’s multiple comparisons test. ANOVA revealed no interaction on genotype and culture condition (ACM) [F (3, 22 = 0.1457; *p* = 0.9314], no effect for genotype [F (1, 22) = 0.03553; *p* = 0.8522] but a significant effect of culture type (ACM) [F (3, 22) = 30.6; *p* = 0.0001]. The data represent a mean of at least three independent experiments. Each experiment consisted of four technical replicates (wells) in which at least three fields were analyzed

Astrocytes secrete a huge variety of factors including proteins, chemokines, cytokines as well as small metabolites such as nucleosides and nucleotides. Proteins can be secreted as individual proteins or within various types of vesicles, such as exosomes. Free proteins may include extracellular matrix components as well as, growth factors, chemokines and cytokines, whereas vesicles can contain membrane proteins as well as RNA [25, 47]. To examine whether macromolecules or small metabolites secreted by astrocytes are responsible for the neuroprotective and synaptogenic effects, C57ACM was fractionated on Amicon cellulose membranes with a 10 kDa molecular weight cutoff. The retained fraction includes proteins and compounds larger than 10 kDa whereas smaller proteins and metabolites are filtered through. Neurons were treated with the solute retained in the filter unit (bigger than 10 kDa, labelled ≥10 kDa) or with the filtered fraction (smaller than 10 kDa, labelled ≤10 kDa). Figure 6 shows that there was no response to the filtrate ≤10 kDa. However, higher numbers of C57 and P301S neurons, comparable to the numbers observed with the full C57ACM, were obtained when cells were cultured with the C57ACM ≥10 kDa fraction (diluted to the original volume to ensure that the effect is not due to the higher concentration of ACM components in the retained fraction), indicating that astrocyte-derived factor(s) supporting neuronal survival in case of C57A are macromolecule(s) rather than small metabolites.

### Thrombospondin 1 involvement in astrocyte-dependent neuroprotection and neurodegeneration

**Fig. 7:**
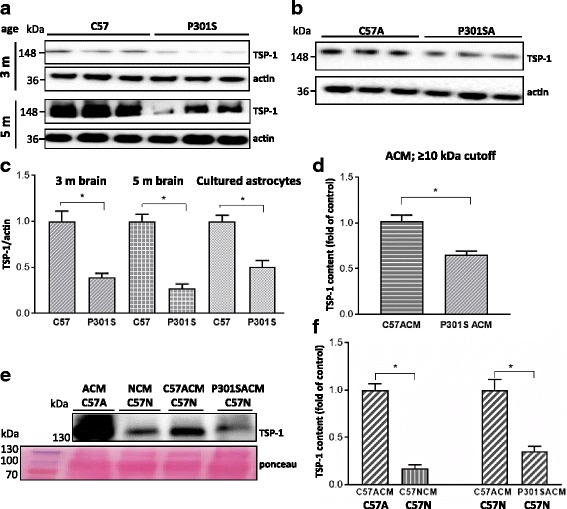
TSP-1 expression is reduced in the superficial cortex and in cultured astrocytes or ACM from P301S mice. **a** Lysates from the superficial cortex of 3 and 5 month-old mice or **b** from astrocytes from 98% pure cultures from 7 day-old mice were analysed for TSP-1 expression by immunoblotting. **c** Quantitative analysis of TSP-1 expression normalised to actin. Values for C57-derived controls are set to 1. *p < 0.05 for these comparisons: C57 3 m vs P301S 3 m; C57 5 m vs P301S 5 mo; C57A vs P301SA. **d** Analysis of ACM from C57A or P301SA after fractionation on an Amicon filter with a 10 kDa cutoff. A significant decrease in TSP-1 was observed in P301SACM compared to C57ACM, *p < 0.05; **e** Representative blot, and **f** quantification of TSP-1.expression in C57ACM, pure C57 neuronal cultures (C57NCM), and C57 neurons co-cultured with C57ACM or P301SACM. Ponceau S staining of ACM blot to show equal loading. *p < 0.05 for these comparisons: C57N + C57ACM vs C57N + P301SACM. C57A released more TSP-1 than C57N, C57A vs C57N. The data represent a mean of three independent experiments performed in triplicates; mean ± S.E.M, statistical analyses performed with Mann–Whitney test

In a preliminary analysis of the proteome of C57ACM and P301SACM, we noted that thrombospondin 1 (TSP-1), a protein heavier than a 10 kDa molecular weight, was reduced in P301SACM compared to C57ACM by about 50%. TSP-1 is an astrocyte-derived regulator of synaptogenesis important for synaptic recovery from brain injury [28] as well as neuron survival [46], and its secretion was impaired in an in vitro amyloid model of Alzheimer’s disease [37]. We therefore examined whether TSP-1 may contribute to the effect on the expression of synaptic markers that we observed following exposure of the neurons to ACM. Figure 7a, c shows that cortical extracts from 3- to 5 month -old P301S tau mice contained 30–40% of the amount of TSP-1 present in the control C57 brain extracts. Similarly, the amount of TSP-1 in astrocytes from 8 day-old mice cultured for 3 weeks was significantly reduced by 50% in P301SA compared to C57A (Fig. 7b, c). C57A released higher amount of TSP-1 than P301SA (Fig. 7d). Furthermore, C57 astrocytes secreted significantly higher amounts of TSP-1 than C57 neurons (Fig. 7e) and this difference in the amount of TSP-1 was also found when ACMs were added to neurons for 24 h (Fig. 7f).

**Fig. 8:**
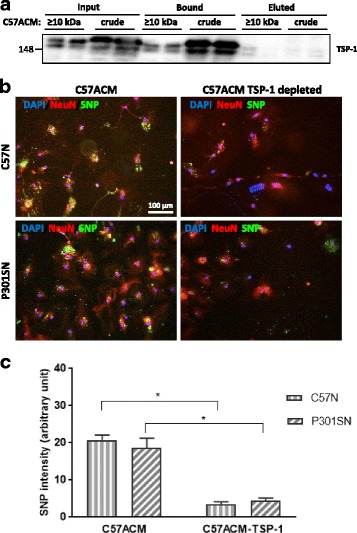
TSP-1 depletion reduces the synaptogenic effects of C57ACM. **a** TSP-1 was immunodepleted from crude C57ACM or from the ≥10 kDa fraction and depletion validated by immunoblotting. Blot showsTSP-1 amounts in input, fraction bound to beads, and eluted supernatant after magnetic separation. **b** Representative pictures of SNP and NeuN immunocytochemistry. Complete ACM and TSP-1-depleted ACM were added to the neuronal cultures for 8 days after which cultures were fixed and immunolabelled with antibodies against SNP and NeuN. **c** Quantification of SNP intensity. For quantification of staining intensity of SNP in the green channel from three slides for each experimental condition were analysed. Results indicate a mean which corresponds in each case to the mean of four fields. *p < 0.05 for these comparisons: C57N + C57ACM vs C57N + C57ACM-TSP-1; P301SN + C57ACM vs P301SN + C57ACM-TSP-1. Values were analysed using Tukey’s multiple comparisons test. ANOVA revealed no interaction between genotype and culture condition (ACM or ACM-TSP-1) [F (1, 12) = 0.9814; *p* = 0.3414] but a significant effect was found for genotype [F (1, 12) = 62.94; *P* < 0.0001] while no effect for culture type [F (1, 12) = 1.476; *p* = 0.2478]

**Fig. 9:**
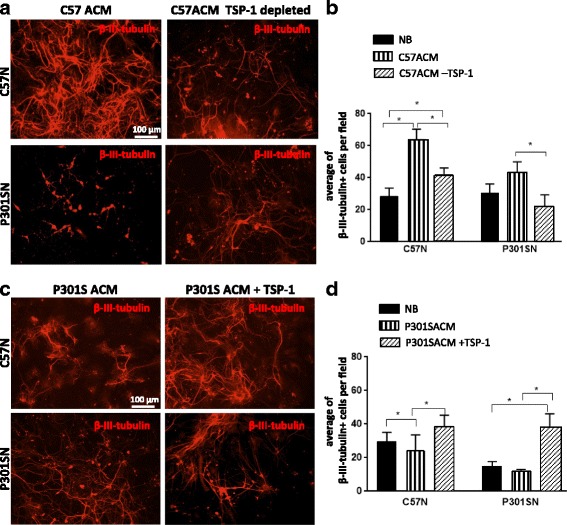
Effect of TSP-1 depletion of C57ACM or supplementation to P301SACM on neuronal survival*.* To study the effect of TSP-1 on neuronal survival C57N and P301SN were cultured in (**a**, **b**) C57ACM or C57ACM depleted of TSP-1 or (**c**, **d**) P301SACM or P301SACM supplemented with TSP-1 for 8 days. **a**, **c** Neuronal cultures were fixed and immunolabelled with anti-β-III-tubulin antibody to determine neuronal number. The data represent a mean of three independent experiments. Each experiment consisted of three technical replicates (wells) in which at least three fields were analyzed. **b** *p < 0.05 for these comparisons: number of neurons in C57N + NB vs C57N + C57ACM; C57N + C57ACM vs C57N + C57ACM-TSP-1; C57N + NB vs C57N + C57ACM-TSP-1; P301SN + C57ACM vs P301SN + C57ACM-TSP-1. **d** *p < 0.05 for these comparisons: number of neurons in C57N + NB vs C57N + P301SACM; C57N + P301SACM vs C57N + P301SACM + TSP-1; P301SN + NB vs P301SN + P301SACM + TSP-1; P301SN + P301SACM vs P301SN + P301SACM + TSP-1. Results were evaluated with Tukey’s multiple comparisons. For experiments where TSP-1 was depleted ANOVA revealed significant interaction on genotype and culture condition (ACM) [F (2, 12) = 18.01; *p* = 0.0002], a significant effect for genotype [F (1, 12) = 22.87; *p* = 0.0004] and significant effect of culture type (ACM) [F (2, 12) = 32.75; *p* = 0.0001]. For experiments where TSP-1 were added, ANOVA revealed no difference between genotype and culture condition (ACM) [F (2, 12) = 2.524; *p* = 0.1217], a significant effect of the genotype [F (1, 12) = 9.39; *p* = 0.0098] and significant effect of culture type (ACM) [F (2, 12) = 32.75; *p* = 0.0001]

To examine whether TSP-1 is implicated in the survival and synaptogenesis of the C57ACM, TSP-1 was immuno-depleted from the C57ACM and the depleted ACM was added to neuronal cultures. Exposure to TSP-1-depleted ACM caused a decline in SNP immunoractivity in both C57N and P301SN (Fig. 8a–c), suggesting that the reduced amount of TSP-1 in the ACM might explain at least in part the loss of synaptic development in the neuronal cultures. Further, immunodepletion of TSP-1 from C57ACM and P301SACM reduced the survival of both C57N and P301SN (Fig. 9a, b). Conversely, the addition of 500 ng recombinant TSP-1 to the P301SACM was sufficient to restore neuronal survival to the levels observed with C57ACM (Fig. 9c, d), suggesting that the reduction in TSP-1 expression in P301SACM may play an important role in the loss of neuronal survival in the P301S transgenic mouse.

## Discussion

Transgenic human P301S tau mice, where tau is expressed specifically in neurons under the control of the Thy1 promoter [1], display progressive tau aggregation and neuron loss with associated astrogliosis in the superficial layers of the cerebral cortex between 2 and 5 month of age [19]. We have previously shown that this neuronal death can be rescued by transplantation of neuronal precursor cell-derived astrocytes from wild type mice [19], implying that the endogenous astrocytes are functionally deficient in P301S tau mice. To determine why transplanted astrocytes were protective we prepared primary co-cultures of postnatal astrocytes and neurons from the cortex of P301S tau transgenic and control mice. Our findings demonstrate that endogenous astrocytes from P301S tau mice are deficient in factors that wildtype astrocytes secrete in order to support neuronal survival and synaptogenesis. Our results thus explain the observation that wildtype astrocytes rescue transgenic P301S tau cortical neurons from death by showing that they express neurosupportive factors which are lacking in P301S-derived astrocytes.

To understand the biochemical basis for these differences we examined the expression of key proteins implicated in astrocyte function. We found an increase in the expression of GFAP and S100β, astrocytic proteins related to glial responses to injury, both in extracts from the cerebral cortex of 3 and 5 month-old P301S mice, extending previous immunohistochemical findings [1, 19], and in primary cultures of astrocytes from P301S tau mice. Correlated with this increase, we found that cultured astrocytes from P301S mice showed enhanced proliferative capacity relative to those from control mice, indicating a cell autonomous memory of a previous injury-like state. Although this does not signify whether these changes are adaptive or maladaptive, they indicate highly coordinated changes in astrocyte behaviour [3, 21]. Our immunoblot analyses also uncovered significant changes in the expression of proteins related to glutamate homeostasis in the superficial cerebral cortex of 3 and 5 month-old P301S tau mice and in primary cultures of astrocytes. Astrocytes secrete glutamate in response to activation, modulate glutamate receptor expression, and remove glutamate from the synaptic cleft by glutamate transporters [2, 4, 49]. This regulation of synaptic glutamate is crucial for normal CNS function, and the sodium-dependent glutamate transport system located peri-synaptically on astrocytes contributes to the regulation of extracellular glutamate levels. Because astrocytes play a major role in control of glutamate homeostasis, we focused on three important regulatory proteins of glutamate metabolism, GS, the main glutamine metabolizing enzyme [34], GLAST and GLT1 [12, 34, 38], the astroglial-specific Na^+^/glutamate transporters. We found a reduced expression of all three proteins in extracts from the superficial cortex of P301S mice, which were also evident in astrocytes cultured from these mice, despite their being expanded for several days ex-vivo.

Decreases in the expression of GLAST and GLT1 were previously reported in astrocytes expressing GFAP/tau mice, wildtype tau or P301L mutant tau [14]. These mice manifested motor deficits before the development of overt tau pathology, which correlated with loss of expression and function of both glial glutamate transporters. Interestingly, there was no difference in effect between the mutant and non-mutant transgenic tau in these mouse models and since tau is not normally expressed in astrocytes, it is not clear how this pathology related to tau toxicity elicited by neurons. Notably, these models are different from our transgenic P301S mice where tau (mRNA and protein) is expressed only in neurons and is not present in astrocytes, indicating that the changes in glutamate transporters in our system must be related to neuron-astrocyte cross talk. In our model, neuronal dysfunction drives the changes similar to a reported mouse model of Parkinson’s disease where disruption of striatal glutamatergic innervation resulted in reduction in both GLT-1 and GLAST protein expression, accompanied by dysfunction of glutamate uptake [16, 23, 31]. A study of a different P301S tau mouse model (where P301S is expressed under the prion promoter) revealed regional changes in glutamate levels that correlated with histological measurements of pathology, such as pathological tau, synapse and neuron loss [13]. Deficits in glutamate neurotransmission and mitochondrial dysfunction were also detected in the frontal cortex and hippocampus of aged 3 × Tg AD mice, which develop beta-amyloid plaques and tau aggregates containing P301L tau [17]. Decreased expression of glutamate-metabolizing enzymes (such as glutamate dehydrogenase and glutamine synthetase protein) in astrocytes were also found in the cerebellum of patients with Alzheimer’s disease [8]. In the 3xTg AD mice, wildtype astrocyte transplantation was reported to improve altered behaviour and this improvement was attributed to increased expression of BDNF [6] but we did not find a significant increase in growth factors following transplantation in our P301S tau mice [19]. A recent study has shown that neuronal activity has a prime role in upregulating gene expression and function of glutamate transporters in astrocytes [20]. Taken together, our results indicate that the glutamatergic system is one of the vulnerable points in the reaction between astrocytes and neurons in brain disease and injury, where astrocytes may fail to prevent glutamate excess and neuronal toxicity through loss of proper glutamate homeostasis.

Both astrocytes from P301S mice co-cultured with neurons, and P301SACM failed to protect neurons from basal cell death whereas C57A or C57ACM enhanced neuron survival. Notably, similar results were obtained using ACM from astrocytes from P301L mice, where tau is expressed under the same neuronal specific Thy1 promoter as in our P301S mice [45]. Hence, the lack of survival support is not tau mouse model-specific nor is it related to a specific tau isoform or MAPT mutation or due to the insertion site of the transgene in the mouse genome but rather is due to the expression of mutant tau and tau pathology development. Although tau filaments and motor pathology develop consistently between 3 and 5 months in the P301S mouse, transgenic tau is expressed from postnatal day 1 and significant signs of altered behavioural function, detected by measuring ultrasound vocalisation (USV) [39], are evident already in newborn mice 3 days postnatal with increased USV maintained up to 7 days [40]. Our findings indicate that astrocytes develop pathological changes due to the exposure to P301S tau-expressing neurons in 7–8 day-old pups but not in 1–2 day-old mice, since we found no difference in neuron survival when neurons were exposed for 8 days to astrocytes or ACM prepared from 1 to -2 day-old P301S tau mice. Although transgenic tau is present in neurons in 1–2 day-old pups, it is possible that either it is not sufficient to induce the astrocytic reaction or that this response takes several days to develop. At both ages, in 1–2 day- or 7 day-old pups no aggregated tau is visible in neurons, indicating that toxic events precede tau filament formation. Hence the development of astrocyte dysfunction appears to relate to the earliest manifestations of neuronal tau toxicity.

Recently, IPSCs-derived astrocytes from Down syndrome (DS) patients were shown to be toxic to neurons but in this case astrocytes, like neurons, bear a trisomy of chromosome 21 [9] whereas MAPT is located on chromosome 17. Similar to our findings, however, the study revealed that DS astroglia exhibited a higher proliferation rate, and expressed higher levels of S100β and GFAP. Furthermore, DS astrocytes contributed to the reduction of neurogenesis of DS NPCs and to the induction of DS neuron death via failure to promote maturation and synapse formation in these cells. Loss of functional synapses is a major neuropathological feature that is well defined in many AD and FTD mice models [10, 32, 41]. In keeping with these results, we observed a significant decline in expression of the synaptic markers PSD95 and synaptophysin upon exposure of neurons to P301SACM. In contrast, exposure to C57ACM enhanced both neuron survival and expression of the two synaptic markers we investigated.

To determine the possible factors involved in astrocyte dysfunction, we sought proteins that are differentially expressed in the ≥10 kDa fractions of the ACM that may be associated with a neuroprotective effect, and thereby focused on TSP-1. TSP-1 is a well defined molecule expressed in postnatal and young adult animal brains and in human cortical astrocytes where it has been shown to promote neuroprotection [5, 30, 54], to increase the number of synapses [11, 24], as well as to accelerate synaptogenesis [50]. Furthermore, TSP-1 has been implicated in neurodegenerative diseases in that addition of amyloid-β peptides, the main components of the amyloid plaques found in the brains of Alzheimer’s patients, caused a significant decline in the release of TSP-1 from primary cultures of astrocytes [37]. We found that P301S astrocytes *in vivo* and *in vitro* produce, and, *in vitro*, release significantly less TSP-1. A similar decline of TSP-1 expression was described in Down Syndrome astroglia pathogenesis [9]. To demonstrate that TSP-1 is a limiting factor in the P301SACM, immune depletion of TSP-1 from C57ACM significantly reduced neuronal survival of C57N and P301SN, whereas supplementation of TSP-1 to P301SACM restored viability, especially that of P301SN. Although we focused on TSP-1, a preliminary analysis of ACMs indicates that it is unlikely that TSP-1 is the only factor that is limiting in P301SACM. A proteomic study of adult symptomatic prion promoter-driven P301S mouse brains identified some differentially expressed proteins in astrocytes, which they propose to have neuroprotective functions [53]. However, the prion promoter may drive expression of tau in astrocytes [29] whereas in our model no tau is expressed in astrocytes. A key question that remains is to find out why and how the expression of these proteins is differentially regulated through neuron-astrocyte interactions.

## Conclusion

The present study reports that C57ACM exhibits neuronal pro-survival properties whereas P301SACM failed to protect neurons from basal cell death. Similar lack of neuronal support by ACM were observed in an independent P301L mouse model, where tau is expressed under the same neuronal specific Thy1 promoter, indicating that our results can be generalized as being a result of tau pathology. This effect of neuronal transgenic tau on endogenous mouse astrocytes develops during the first week of life in the brain of P301S and P301L mice, given that the lack of neuronal support observed in astrocytes from 7 day-old pups is not present in astrocytes from 1 to 2 day-old mice. Although transgenic tau is present in 1–2 day-old pups either its amount is not sufficient to induce the astrocytic reaction or this can take some days to develop. At both ages, in 1–2 day or 7 day-old pups no filamentous aggregated tau is visible in neurons suggesting that the toxic event can precede tau filament formation. Furthermore, we demonstrate that endogenous astrocytes derived from 7 day-old human P301S tau mice lack key molecules that regulate glutamate homeostasis, and support neuronal survival and synaptogenesis. Understanding the molecular events of astrocyte induced dysfunction will lead to a better understanding of the disease process, while the result obtained for TSP-1 may have promising implications for the development of future treatment strategies for neurodegenerative disorders, such as tauopathies.

## Additional file

Additional file 1: Figure S1. a. Immunofluorescent staining for human P301S tau (HT7, green) and GFAP (astrocytes, red). Brain secDons (25 μm) from 9 day (top row) and 5 m (boLom row) P301S mice showing no overlap. b. The blot shows that P301S tau is not expressed in cultured astrocytes from 9 day old C57 (C), P301S (PS) or P301L (PL) pups (middle panel, 2 independent 8-­‐day cultures); the right hand two lanes show tau expression in brain extracts from 5 m C57 and 5 m P301S mice run on the same blot as negaDve and posiDve controls. Top panel, Ponceau; boLom panel, blot reprobed for β-actin (without stripping). mw, marker lane. (PDF 1.47 mb)

## Abbreviations

ACM: Astrocyte conditioned medium
AD: Alzheimer’s disease
CBD: Corticobasal degeneration
DS: Down syndrome
FTDP-17T: Frontotemporal dementia and parkinsonism linked to chromosome 17
GFAP: Glial fibrillary acidic protein
GLAST: Glial high-affinity glutamate aspartate transporter
GLT1: Glial glutamate transporter
GS: Glutamine synthetase
MAPT: Microtubule associated protein tau
NCM: Neuronal conditioned medium
NPC: Neuron precursor cell
PiD: Pick’s disease
PSP: Progressive supranuclear palsy
SNP: Synaptophysin
Tg: Transgenic
TSP-1: Thrombospondin 1
USV: Ultrasound vocalisation
