# Supplementary material for: Astrocytes in mouse models of tauopathies acquire early deficits and lose neurosupportive functions
Source: Acta Neuropathol Commun. 2017 Nov 29;5:89. doi: 10.1186/s40478-017-0478-9 (PMC6389177; doi:10.1186/s40478-017-0478-9)
Supplement: Supplementary file 1 — a. Immunofluorescent staining for human P301S tau (HT7, green) and GFAP (astrocytes, red). Brain secDons (25 μm) from 9 day (top row) and 5 m (boLom row) P301S mice showing no overlap. b. The blot shows that P301S tau is not expressed in cultured astrocytes from 9 day old C57 (C), P301S (PS) or P301L (PL) pups (middle panel, 2 independent 8-­‐day cultures); the right hand two lanes show tau expression in brain extracts from 5 m C57 and 5 m P301S mice run on the same blot as negaDve and posiDve controls. Top panel, Ponceau; boLom panel, blot reprobed for β-actin (without stripping). mw, marker lane. (PDF 1.47 mb) [file 40478_2017_478_MOESM1_ESM.pdf]

a

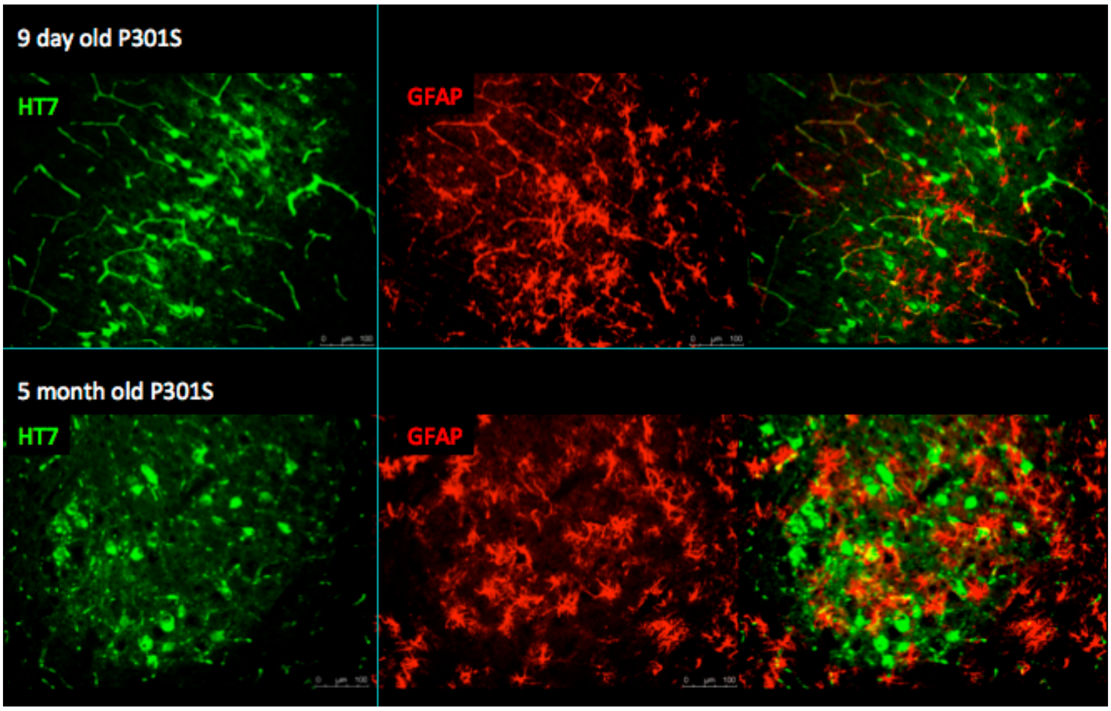

b

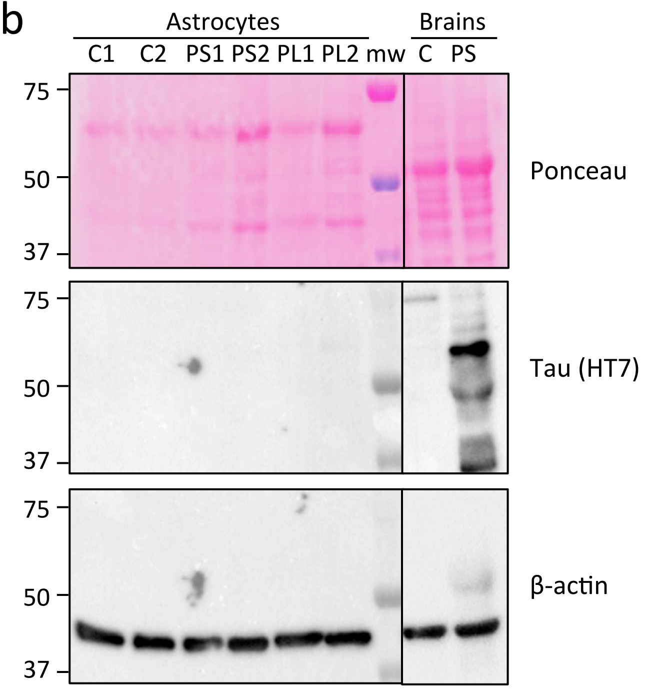

Supplementary Fig. 1. a. Immunofluorescent staining for human P301S tau (HT7, green) and GFAP (astrocytes, red). Brain sections (25 µm) from 9 day (top row) and 5 m (bottom row) P301S mice showing no overlap. **b.** The blot shows that P301S tau is not expressed in cultured astrocytes from 9 day old C57 (C), P301S (PS) or P301L (PL) pups (middle panel, 2 independent 8-day cultures); the right hand two lanes show tau expression in brain extracts from 5 m C57 and 5 m P301S mice run on the same blot as negative and positive controls. Top panel, Ponceau; bottom panel, blot reprobed for β-actin (without stripping). mw, marker lane.
